# Identifying Research Trends and Gaps in the Context of COVID-19

**DOI:** 10.3390/ijerph17103370

**Published:** 2020-05-12

**Authors:** Hongyue Zhang, Rajib Shaw

**Affiliations:** 1 Ocean College, Minjiang University, Fujian 350108, China; 2589@mju.edu.cn; 2 Graduate School of Media and Governance, Keio University, Fujisawa 252-088, Japan

**Keywords:** coronavirus research trends, COVID-19, public health and disaster risk reduction, social science research, public health preparedness, mitigation, response and recover, World Health Organization research roadmap

## Abstract

The COVID-19 pandemic has affected the world in different ways. Not only are people’s lives and livelihoods affected, but the virus has also affected people’s lifestyles. In the research sector, there have been significant changes, and new research is coming very strongly in the related fields of virology and epidemiology. Similar trends were observed after the Severe Acute Respiratory Syndrome Coronavirus (SARS-CoV) and Middle East Respiratory Syndrome Coronavirus (MERS-CoV) episodes of 2003 and 2012, respectively. Analyzing 20 years of published scientific papers, this article points out the highlights of coronavirus-related research. Significant progress is observed in the past research related to virology, epidemiology, infectious diseases among others. However, in research linked to public health, its governance, technology, and risk communication there seem to be gap areas. Although the World Health Organization (WHO) global research road map has identified social science-related research as a priority area, more focus needs to be given in the upcoming days for multi, cross and trans-disciplinary research related to public health and disaster risk reduction.

## 1. Introduction

The coronavirus disease COVID-19 was declared a pandemic by the World Health Organization (WHO) on the 11th of March 2020, within less than three months after its first report in Wuhan, China in late December 2019 [1]. The numbers (infected people, death, number of new cases, among others) are increasing on a daily basis. From China, the hotspots shifted first to Europe and then to the USA. Many countries have declared indefinite to time-bound lock downs, with severe restrictions on in-country as well as international travel. Tremendous pressure is mounting on countries’ health care systems and health care professionals are also becoming direct victim of the virus in many cases, with reported deaths in several countries.

Three major characteristics of COVID-19 are: (1) high rate of spread, (2) aged and low immunity people are more vulnerable to be infected, and (3) differential recovery rates in different countries [2]. While the urgent need is to develop the appropriate curative medicines and vaccines, there are different types of ongoing research on this topic. Pandemic risk is not just a medical issue, but has strong socio-economic, behavioral, psycho-social, governance and technological implications. Of course, virology, medical science, epidemiology are the core for addressing the key issue. However, these also need to be linked to other issues. Governance and decision-making related to pandemic risk are important points, which need to be discussed more.

Incorporation of pandemic/biological hazard risk in the Sendai Framework of disaster risk reduction is important [3], and it is mentioned that the global framework can be well linked with the health emergency and disaster risk management (H-EDRM) concept. We also see different governance-related decisions in East Asia which were effective in different stages of the pandemic [2]. Similarly, different innovative technologies have been used for pandemic response in different countries. A comprehensive review of the technology and its innovative use for serving different types of services during lock-down is yet to be done. People’s behavior is one of the key aspects of pandemic response. Risk perception, understanding and communication are also linked to behavior change. New research would come out on the behavior as well as social changes during pandemics. There are also tremendous economic implications of the pandemic, which can be observed at global, regional, national and local levels. Some of the economic issues are also linked to the local social issues, especially for micro-, small- and medium-enterprises (MSMEs).

Keeping this in mind, this paper makes a modest attempt to analyze past and current research trends related to coronavirus over a period of 20 years. Detailed analysis and discussions are provided from the papers published in last three months. The analysis shows some gaps and issues, which needs to be linked to the global research road map of WHO [4].

## 2. WHO Research Road Map

On 30 January 2020, following the recommendations of the Emergency Committee, the WHO Director General declared that the COVID-19 outbreak constituted a Public Health Emergency of International Concern (PHEIC). World experts on COVID-19 met at the WHO headquarters in Geneva from the 11 to 12 February 2020, to assess the current level of knowledge about the new virus, agree on critical research questions that needed to be answered urgently and ways to work together to accelerate and fund priority research that can contribute to curtail this outbreak and prepare for future outbreaks [4]. The global imperative for the research community is to maintain a high-level discussion platform which enables consensus on strategic directions, nurtures scientific collaborations and, supports optimal and rapid research to address crucial gaps, without duplication of efforts. Experts identified key knowledge gaps, and research priorities and shared scientific data on ongoing research, thereby accelerating the generation of critical scientific information to contribute to the control the COVID-19 emergency. Eight immediate research actions were proposed as follows:

- Mobilize research on rapid point of care diagnostics for use at the community level
- Immediately assess available data to learn what standard of care approaches from China and elsewhere are the most effective.
- Evaluate as fast as possible the effect of adjunctive and supportive therapies.
- Optimize use of protective equipment and other infection prevention and control measures in health care and community settings.
- Review all evidence available to identify animal host(s), to prevent continued spillover and to better understand the virus transmissibility in different contexts over time, the severity of disease and who is more susceptible to infection.
- Accelerate the evaluation of investigational therapeutics and vaccine using “Master Protocols”.
- Maintain a high degree of communication and interaction among funders so that critical research is implemented.
- Broadly and rapidly share virus materials, clinical samples and data for immediate public health purposes.

The target of the WHO Research Road Map was: (1) to ensure that the affected people gets proper diagnosis and optimum care, and (2) to support research priorities that lead to development of global research platform. A cross-cutting, inter-disciplinary approach was suggested which looks at the ethical aspects, as well as practical implications with international solidarity.

Nine specific thematic areas were suggested as follows:

- Virus: natural history, transmission and diagnostics.
- Animal and environmental research on the virus origin, and management measures at the human-animal interface.
- Epidemiological studies.
- Clinical characterization and management.
- Infection prevention and control, including health care workers’ protection.
- Candidate therapeutics research and development
- Candidate vaccines research development
- Ethical considerations for research.
- Social sciences in the outbreak response.

Thus, broadly speaking there are three categories of research areas: (1) Science and technology (related to the virus, animals, the environment and epidemiology); (2) Governance and management (clinical management, health care worker’s protection, ethical issues); (3) Social and behavior issues (people and citizen’s response behavior).

In their recent book, [5] has argued the challenges and gaps in H-EDRM. The WHO pointed out in 2019 that H-EDRM remains as a fragmented nascent field, and needs to be developed as a coherent enterprise. Key challenges include non-alignment of research tools, lack of a strategic overarching research agenda, sub-optimal development of multisectoral and interdisciplinary approaches, absence of the science-policy-practice nexus, deficiency in standardized terminology, and meagre coordination among stakeholders [6]. It is interesting to note that the road map of WHO for COVID-19 makes no mention of H-EDRM. This issue will be discussed later.

## 3. Methodology

To understand the research trends, the Web of Science core collection database was used to search for SCIE and SSCI papers related to coronavirus published in 2000–2019. The search formula was defined as “TS = Coronavirus”, and the language was set to English. In November 2002 Severe Acute Respiratory Syndrome Coronavirus (SARS-CoV) broke out in Guangdong, China, and became an acute epidemic in 2003. Middle East Respiratory Syndrome Coronavirus (MERS-CoV) is reported in Saudi Arabia in 2012 and has since spread to different countries.

In this analysis, twenty years (2000 to 2019) have been broken into five time slices: 2000–2003, 2004–2007, 2008–2011, 2012–2015, and 2016–2019. See Table 1 for the main information about the publication situation in each time period.

In order to analyze the evolution characteristics of coronavirus research topics over time, a word cloud was used to reflect the dominant topics during each period. A ‘word cloud’ is a visual representation of word frequency. The more commonly a term appears within the text being analyzed, the larger the word appears in the generated image. Word clouds are increasingly being employed as a simple tool to identify the focus of written material [7]. Also, co-word analysis [8,9,10] was used to construct a co-occurrence map to reveal the research hotspots and evolution of each time slice. Co-word analysis is used in a longitudinal framework which allows us to analyze and track the evolution of a research field along consecutive time periods [8]. Additionally, it develops a performance analysis of specific themes using different basic bibliometric indicators.

Author keywords are a list of terms that authors believe best represent the content of their papers. In the following study, the hot topics of each time slice are visualized by building the word cloud of author keywords. Since titles and abstracts can interpret the contents of paper more comprehensively than keywords, terms extracted from titles and abstracts are used to construct the co-word map by VOSviewer and the topic clusters of each time slice are analyzed based on the co-word map.

## 4. Research Trends Until 2019

### 4.1. Time Period: 2000–2003

**Figure 1:**
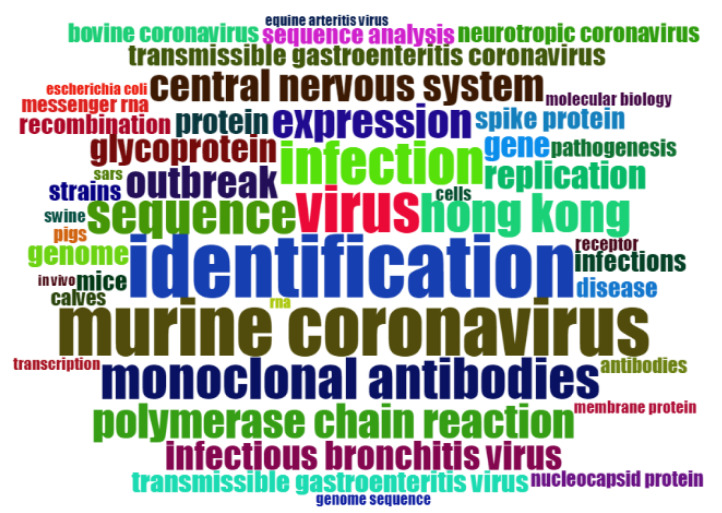
Keywords Cloud Map of 2000–2003.

From 2000 to 2003, the frequency of SARS occurrences was 141, and the total cited frequency was 11,723. That is to say, the SARS research in 2003 has received widespread attention worldwide (Figure 1). In addition, the research frequency of HEPATITIS (a type of disease) during this period is also very high, 65 times, and the total cited frequency is 2544. The research on HEPATITIS is basically based on Mouse. From the statistical results, the hot topics in 2000–2003 shown in Table 2.

There are two papers cited over 150 times during 2000–2003, one is written by Luo et al. (2000) published in *Journal of Virology*, titled “Retargeting of coronavirus by substitution of the spike glycoprotein ectodomain: crossing the host cell species barrier”. The other is written by Lane et al. (2000) also published in *Journal of Virology*, titled “A central role for CD4(+)T cells and RANTES in virus-induced central nervous system inflammation and demyelination”.

**Figure 2:**
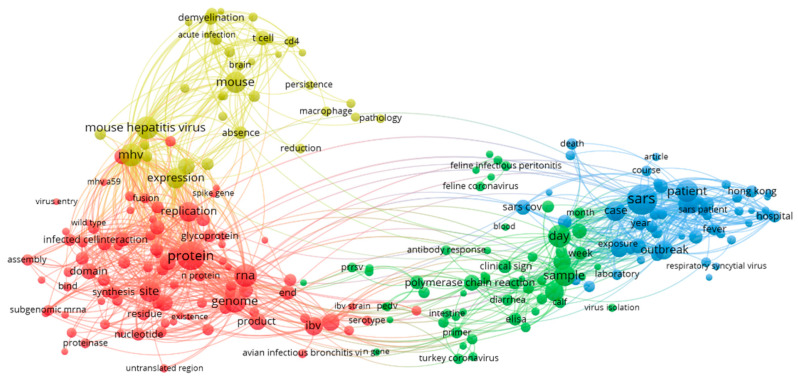
2000–2003 term co-occurrence map.

VOSViewer is adopted to construct the terms cooccurrence map. Terms are extracted from the title and abstract fields, the minimum number of occurrences of a term is set as 10, and a relevance score is calculated for each term. Based on the score, the 60% most relevant terms are selected to build the cooccurrence map (Figure 2). Blue clusters are studies of SARS cases, and the red clusters are studies of proteins and genes. The yellow clusters are studies of mouse hepatitis; and the green clusters are studies of antibody chain reactions. The analysis shows a strong presence of red clusters (protein and gene). The inter-disciplinary nature of the research, which is required, may not be that strong.

### 4.2. Time Period: 2004–2007

**Figure 3:**
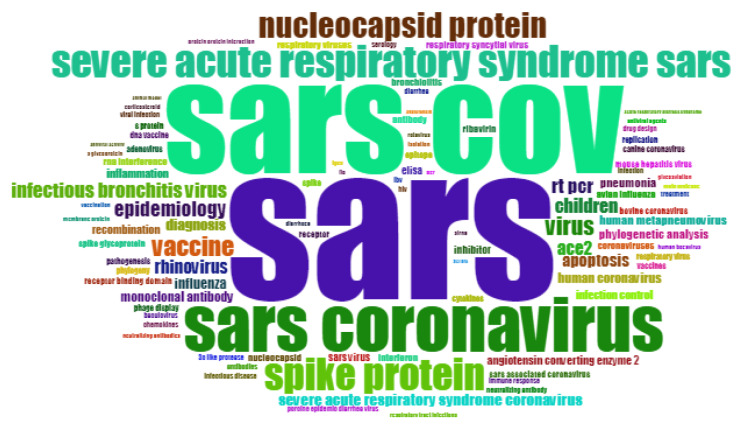
Keywords cloud map of 2004–2007.

From 2004 to 2007, SARS has become a research focus and focus, with 977 occurrences and 29,584 total citations (Figure 3). 

“Protein” occurred 490 times with 14,994 citations, of which “spike protein” appeared 184 times and was cited 7155 times. In addition, “patients” appear 137 times, with 5108 citations, “nucleocapsid” appears 120 times, and its frequency of citations reached 3622 times. The hot topics in 2004–2007 extracted from the statistical results are shown in Table 3. It is noted that there are seven articles that were cited more than 500 times between 2004 and 2007. The information is shown in the Table 4.

**Figure 4:**
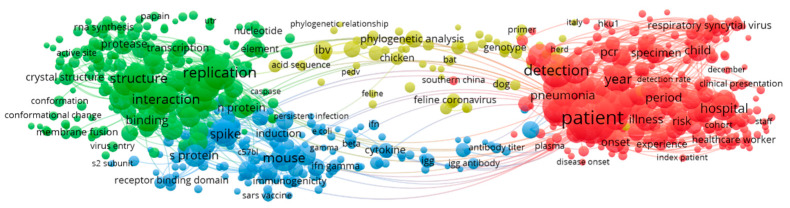
2004–2007 Term Cooccurrence Map.

The term cooccurrence map are shown in Figure 4. Red clusters are researches on hospital case detection. The green cluster is the study of virus structure and replication and interaction after invading the human body. Yellow clusters are for phylogenetic analysis; and blue clusters are the first-body binding domains and immunodeficiency studies in mice. There is a definite high concentration of red cluster (hospital case detection), followed by green (virus structure and replication and interaction).

### 4.3. Time Period: 2008–2011

**Figure 5:**
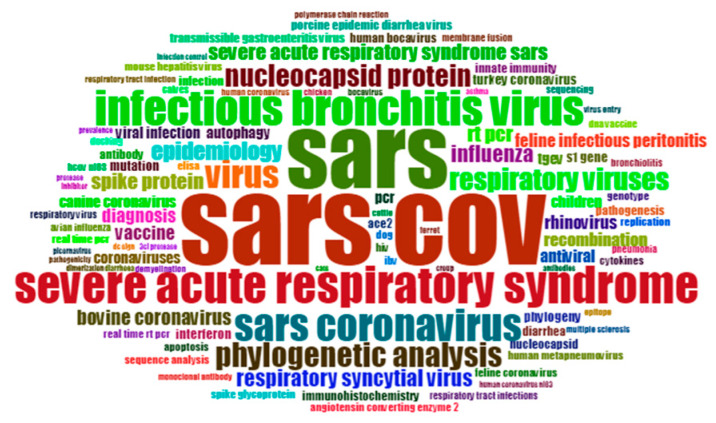
Keywords cloud map of 2008–2011.

From 2008 to 2011, research on SARS did not decrease, with 332 occurrences and a total of 6961 citations. In addition, “severe acute respiratory syndrome” appeared more than 200 times, with a total of 5000 citations; “bronchitis” appeared 98 times, with a total of 2933 citations; “children” appeared 69 times, and a total of 2933 citations (Figure 5). The topic trends of 2008–2011 are illustrated in Table 5. During 2008–2011, there were four hot papers with over 400 citations, and the information is shown in Table 6.

**Figure 6:**
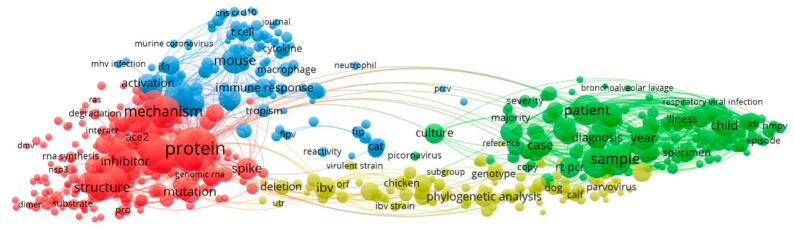
2008–2011 term co-occurrence map.

The term cooccurrence map of 2008–2011 publications is generated in Figure 6. The red cluster is the study of protein structure and mechanism, and blue clusters are used to study the immune response to viruses in mice and anchors. Green cluster is the study of diagnosis and treatment of cases and children, and the yellow cluster is a phylogenetic analysis of epidemic dysentery using chickens and dogs. Here, a strong concentration is found in green cluster (diagnosis and treatment of cases and children), followed by red cluster (protein structure and mechanism).

### 4.4. Time Period: 2012–2015

**Figure 7:**
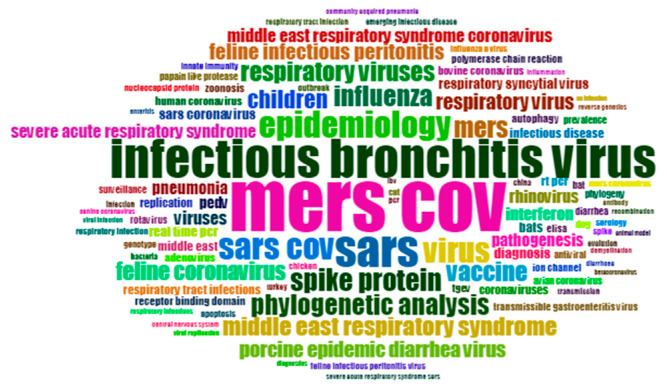
Keywords cloud map of 2012–2015.

From 2012 to 2015, MERS-COV is the most prevalent topic, with 320 occurrences and cited 13,882 times. “SARS” occurred 161 times and was cited 3359 times. “Infectious bronchitis virus” occurred in 113 papers with 1815 citations (Figure 7). During this period, keywords including “epidemiology”, “respiratory viruses”, “spike protein”, “influenza”, “phylogenetic analysis”, “vaccine” and “children” are also hot topics, with over 30 occurrences. The trending topics of each year during 2012–2015 are shown in Table 7. During 2012–2015, there are four papers which were cited over 400 times, (Table 8).

**Figure 8:**
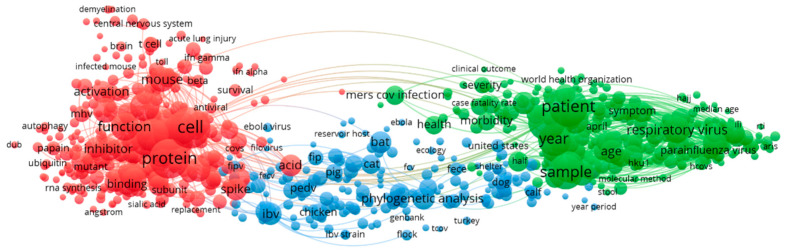
2012–2015 term co-occurrence map.

The terms extracted from title and abstracts of 2012–2015 publications on coronavirus are used to build the cooccurrence map shown in Figure 8. Red clusters are experiments on mouse proteins and RNA synthesis in mice and blue clusters are phylogenetic analysis of infectious bronchial virus (IBV), porcine epidemic virus (PEDV), cats, dogs, and bats. Green clusters are case studies. It was interesting to note that the green clusters have more concentration, which are on case studies.

### 4.5. Time Period: 2016–2019

**Figure 9:**
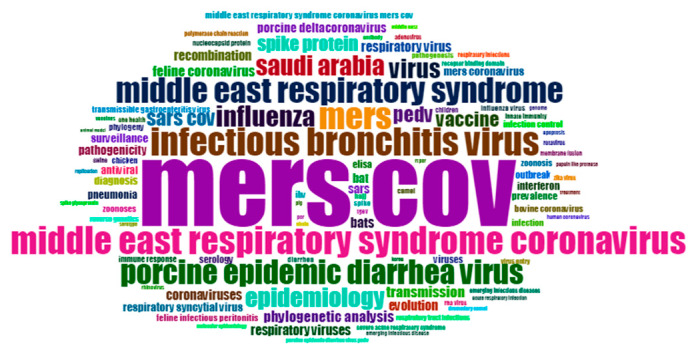
Keywords cloud map of 2016–2019.

During 2016–2019, it can be seen from Figure 9 that MERS-COV is still the hot issue, occurring 493 times, and cited 4065 times according to the statistical results. “Infectious bronchitis virus” occurred 151 times with 856 citations. “Porcine epidemic diarrhea virus” is also a hot topic in this period, with 74 citations. The most frequent topics during 2016–2019 also included “Saudi Arabia”, “epidemiology”, “pedv”, “SARSCov”, “vaccine”, “spike protein” and “phylogenetic analysis”, with over 35 occurrences, respectively. The trending topics of each year during 2016–2019 are shown in Table 9. There are five papers cited over 110 times during 2016–2019 (refer to Table 10).

**Figure 10:**
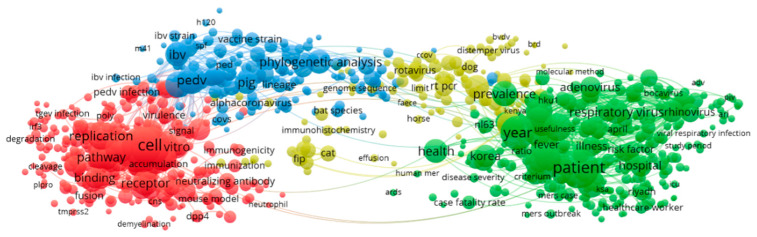
2016–2019 term co-occurrence map.

The term cooccurrence map of 2016–2019 is shown in Figure 10. The red clusters are studies on the expression and replication of viral enzymes in recipient cells, human immunodeficiency virus, among others. Green clusters are research on patients, hospitals, children and health. Blue clusters are studies of vaccines, virus strains, and the yellow clusters are researches using polymerase chain reaction by animals. Here also, important issue is the increase of green clusters which are on patients, hospitals, children and health.

### 4.6. Summary of Research Trends: 2000–2019

**Figure 11:**
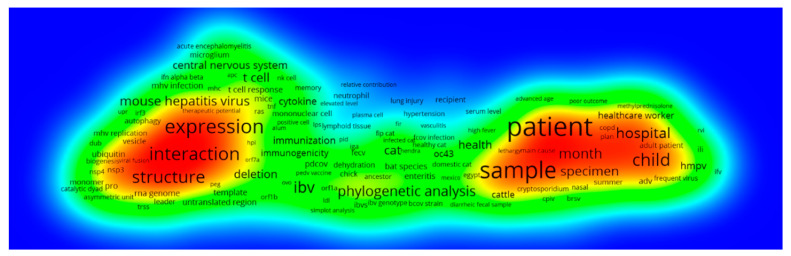
2000–2019 term co-occurrence density map.

From 2000 to 2019, there are two main research hotspots: one is about the structure, expression and interaction of the coronavirus after entering the human body; the other is about the case of patients, including children (Figure 11). Studies on gene expression, interactions and structural changes after the virus invades the human body are mainly focused on the development of antibodies and vaccines, animal experiments, virus mutations and activity changes; Medical research on patients with coronaviruses is mainly focused on patient immune response, diagnosis and treatment.

From the time series analysis, it is observed that: public safety incidents affected scientific output and research topics, as observed after SARS and MERS. This may be an obvious increase in terms of focus on research topics. In addition to SARS and MERS, there are a few other types of coronavirus research. Whether these viruses will have a profound impact on humans needs further study. Table 11 shows that in the five time periods, research on patients with coronavirus has been a hot spot, and many scholars have conducted research on patients’ symptoms and diagnostic methods, as well as cases of different ages, especially children; In the field of biomedicine, mice are used as test objects, focus on the interaction with cells and virus replication after the virus enters the organism. Spike protein and structural changes of virus, immune reaction, T-cell study as well as antibody development are hot topics after SARS outbreak after 2003. It is worth noting that the study of phylogenetic analysis based on spike protein sequences has become a hot spot in 2012–2019. In the study of coronavirus from 2003 to 2019, in addition to SARS and MERS, IBV, PEDV and mouse hepatitis have also been studied by many scholars.

## 5. Research Trend in 2020

The same methodology was used for 2020 analysis as well. The Web of Science core collection database was used to search SCIE and SSCI papers related to coronavirus published since 2020. The search formula was defined as “TS = Coronavirus” and the language was set to English. The main information of the publication is shown in Table 12.

**Figure 12:**
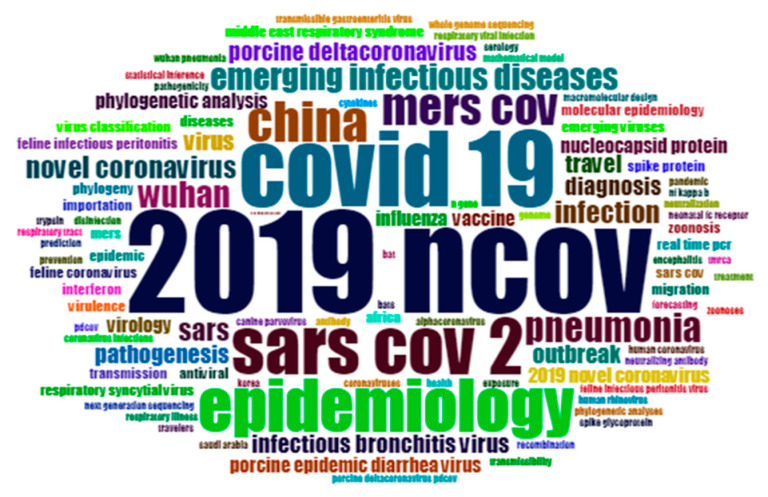
Keywords cloud map of 2020.

Since the outbreak of the COVID-19 in late 2019, a total of 384 papers have been published. Epidemiological knowledge is adopted to study infection of the virus. Thus, epidemiology and emerging infection diseases become two prominent key words after the names of the virus like 2019ncov, covid19, SARScov2 among others. It is to be emphasized here that in several virology/epidemiology papers, SARS-COV2 refers to the virus which caused the disease, and the disease itself is referred as COVID-19 or 2019cov. In order to analyze the trend of COVID-19 research, Keyword Plus (provided by Web of Science) and author keywords are both adopted to build the trend map (Figure 12) based on the logarithm of keyword occurrences. It should be noted that as keyword plus illustrated, besides acute respiratory syndrome, MERS, replication and expression, a small cluster including pathogenicity, influenza, diarrhea, respiratory syndrome, RIG-I, syncytial virus and crystal-structure shows an aggregate status. Author keywords reveal that studies on virus, pathogenesis, outbreak, travel and porcine delta coronavirus are linked together, and shows a continuing trend. Five papers cited over 40 times are shown in Table 13.

**Figure 13:**
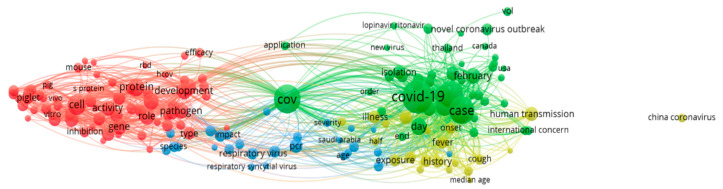
2020 term co-occurrence map.

Figure 13 shows the term cooccurrence map. Red clusters represent research on proteins and cellular genes. Green clusters represent research on COVID-19 case outbreaks. Yellow clusters represent studies of infection history and symptoms, while blue clusters represent research on respiratory viruses.

As can be seen from the green cluster, the research on COVID-19 has attracted international attention. The author keywords reveal that besides China, other countries include the United States, Canada and Thailand. Fever and cough are the main symptoms of COVID-19, so “fever” and “cough” appear more frequently in the author keywords. In addition, population migration has played a huge role in spreading the virus, so isolation is an effective means to limit the spread of the virus. The terms mouse and pig in the red cluster are receptors of coronavirus and seem to be used for medical tests. Other keywords also include “cell”, “protein”, “gene” and “pathogen”.

From the currently published papers, it is observed that the research on COVID-19 mainly includes epidemiological characteristics, virus traceability studies, early transmission, mode and modeling analysis of COVID-19 predicting the spread and trend of the epidemic, clinical characteristics of COVID-19, diagnosis and treatment also attracted wide attention. The development of antibodies and vaccines will be of great concern. As of 6 May 2020, the number of people infected with COVID-19 has exceeded 3.8 million globally. The global and local economies are highly affected, and socio-economically vulnerable people like daily wedger are at high risk.

## 6. Discussion and Way Forward

As per [11], experts recognized that an important amount of information is available just three months into the outbreak, there are still concerns about knowledge gaps and lack of clear evidence to support some interventions. The importance of strengthening capacity was highlighted in the research roadmap meeting in February 2020. Integration of research activities in the response to outbreaks and the lessons learnt on SARS, Ebola, Lassa fever, and Nipah have led to a prompt research response now. Participants emphasized that as we mobilize the research community for COVID-19, concerted efforts should be made to facilitate the sustainment of this capacity to support other ongoing or future outbreaks across the world. On the 26th of March, WHO issued six prioritized strategies, to be undertaken by governments to cope with the pandemic. The strategies were as follow: (1) Expand, train and deploy health-care workers; (2) Implement systems to find suspected cases; (3) Ramp up production of tests and increase availability; (4) Identify facilities that can be transformed into coronavirus health centers; (5) Develop plans to quarantine cases; and (6) Refocus governments on suppressing the virus [11]. The document also identified eight knowledge gaps as follows: (1) human animal interface, (2) clinical consideration, (3) vaccine, (4) behavior and education, (5) transmission, (6) therapeutics, (7) healthcare workers, and (8) ethical considerations.

In its analysis, [3] calls for an international protocol of pandemic response, where similar standards are processes are maintained. That needs more research and continued international dialogue. Governance become a key challenge in many countries, and not that much research is conducted on pandemic governance and needs to be considered as a core research gap. In the context of governance the H-EDRM [12], which was described earlier can provide a basic framework. Incorporation of pandemic risk and biological hazards need to be incorporated in the Sendai Framework national implementation in respective countries, which seems to be a gap area. India is one exception, where the National Disaster Management Act (Law) was enacted country wise to respond to COVID-19 for the first time since the promulgation of the law in 2005. Thus, there seems to be a strong gap of incorporation of biological hazards in disaster response, recovery and long-term preparedness. New research is required in the areas of supply chain management, business continuity planning (BCP) and short to medium term response and recovery planning. We also did not notice much research on the risk assessment methodologies, which is an integral part of the disaster response and risk reduction. Multi-disciplinary research incorporating public health, disaster risk reduction, economics of pandemics, social psychology, anthropology, sociology, psychology and ecology are required.

Social innovation in long term care can be seen as the potential option for addressing COVID-10 from the analysis of the Italy case [13]. Social innovation key factors in Italian Long Term Care are listed as follows: (1) coordination/integration in public health care, (2) design to meet target group needs, (3) framework/structural conditions, (4) funding, (5) leadership and governance, (6) local community focus, (7) specificity of LTC, (8) network, (9) sustainability, and (10) workforce. These elements need to be integrated properly to develop new social innovation in long term care. This area needs a strong focus along with development of other innovative and emerging technologies, linked to social innovation.

The International Network of Government Science Advice (INGSA) in its COVID-19 theme has identified sixteen different topics: data visualization/tracking tool, research/education tools, risk communication/preparedness, economics, social resilience/social cohesion, international cooperation/science diplomacy, science communication/trust, national governance, ethics/social science, policy development, role of digital technologies, sub-national governance, global south COVID response, emergencies and mental health, regulation and emergency response, and human rights issues [14].

The key issue emerging from this literature analysis is that over a period of 20 years, different types of epidemic diseases have prompted innovative research on epidemiology, virology, disease infection, vaccine, impacts on different age groups among others. However, an epidemic or pandemic response is not just a health issue, it is very much a disaster response. Thus, basic disaster response framework needs to be adopted and followed. More research is required for new research on biological hazard response, role of different types of technology, governance mechanisms, risk communication, people’s behavior and citizen participation. On the public health side, public health preparedness, implementation of H-EDRM frameworks, experience sharing through open access data sharing and publication is required to reduce the north south divide of knowledge management [15]. Research communication, and role of media is another important issue, which is often not properly researched. As argued in [1], the COVID-19 response as “infodemic”, the role of right information through reliable sources and at right time is very important. Infodemic was the term used by WHO to highlight the importance of right and timely information during pandemic. This is the core of risk communication for invisible disasters, where trust on information becomes a core issue. Research on early recovery planning is also urgently required.

As it is said that earthquake problem cannot be solved by seismologist or earthquake engineers, it needs a wider expertise of planner, social scientist, economists among others. Similarly, the pandemic risk reduction and response needs expertise beyond health professionals. In future we hope to see more inter-, trans- and multi-disciplinary approaches in health risk management, pandemic response, disaster risk reduction.

## 7. Conclusions

Analysis of research papers with keywords “Coronavirus” over last 20 years shows that there has been an increase in the COVID related research after major Coronavirus spread like SARS, MERS etc. Most of the research focus on virology, immunology epidemiology etc., however there is little research on linking biological hazards (including pandemic) to disaster response, covering holistic approach of response. While H-EDRM framework provides an opportunity to the integration of public health and disaster risk reduction, response and recovery, new research needs to focus on different aspects of pandemic response, recovery and long-term development. COVID-19, being an infodemic, information, risk communication, citizen behavior are areas which needs additional research. 

## Figures and Tables

**Table 1:** Main information of the relevant coronavirus publications between the year of 2000 and 2019.

Time Period	2000–2003	2004–2007	2008–2011	2012–2015	2016–2019
Number of Publications	848	2706	1824	2446	2786
Source journals	226	599	511	585	645
Author keywords	881	2911	2881	3278	4610
Average citations per documents	57.32	39.96	31.24	26.41	7.19
Authors of single-authored documents	56	131	69	133	91
Authors of multi-authored documents	2624	8944	6940	9617	11,886
Collaboration Index	3.36	3.51	3.97	4.22	4.43

**Table 2:** Items and frequency of hot keywords between 2000–2003.

Item	Frequency	Year
CORONAVIRUS	168	2003
MOUSE HEPATITIS-VIRUS	137	2001
IDENTIFICATION	90	2003
MURINE CORONAVIRUS	82	2001
ACUTE RESPIRATORY SYNDROME	69	2003
VIRUS	67	2002
INFECTION	62	2001
MONOCLONAL-ANTIBODIES	59	2001
SEQUENCE	58	2002
HONG-KONG	55	2003
EXPRESSION	49	2002
OUTBREAK	46	2003
CENTRAL-NERVOUS-SYSTEM	45	2001
GLYCOPROTEIN	40	2002
PROTEIN	35	2002
RESPIRATORY SYNCYTIAL VIRUS	10	2000
MESSENGER-RNAS	10	2000
PACKAGING SIGNAL	7	2000
RECOMBINANT VACCINIA VIRUS	7	2000
BROME MOSAIC-VIRUS	6	2000
MURINE CORONAVIRUSES	6	2000
MAMMALIAN-CELLS	6	2000

**Table 3:** Items and frequency of hot keywords during 2004–2007.

Item	Frequency	Year
INFECTION CONTROL	12	2004
TREATMENT	8	2004
CHEMOKINES	8	2004
CORTICOSTEROID	7	2004
ZOONOSIS	6	2004
TURKEY CORONAVIRUS	6	2004
MULTIPLE SCLEROSIS	6	2004
S GLYCOPROTEIN	6	2004
MOUSE	6	2004
QUARANTINE	6	2004
CORONAVIRUS	242	2005
SARS	212	2005
SEVERE ACUTE RESPIRATORY SYNDROME	171	2005
SARS CORONAVIRUS	64	2005
SEVERE ACUTE RESPIRATORY SYNDROME (SARS)	47	2005
SARS-COV	150	2006
SPIKE PROTEIN	47	2006
NUCLEOCAPSID PROTEIN	38	2006
VIRUS	27	2006
EPIDEMIOLOGY	23	2006
INFECTIOUS BRONCHITIS VIRUS	23	2006
RESPIRATORY VIRUS	9	2007
ADENOVIRUS	9	2007
PORCINE EPIDEMIC DIARRHEA VIRUS	8	2007
HUMAN BOCAVIRUS	7	2007
PCR	7	2007
TGEV	7	2007
GLYCOSYLATION	7	2007

**Table 4:** Top cited papers during 2004–2007.

Author Name	Year	Publication Journal	Title	GCS
Allander T. et al.	2005	*Proc. Nat. Acad. Sci. USA*	Cloning of a human parvovirus by molecular screening of respiratory tract samples	1034
Li WD et al	2005	*Science*	Bats are natural reservoirs of SARS-like coronaviruses	861
van der Hoek L	2004	*Nat. Med.*	Identification of a new human coronavirus	756
Calisher CH	2006	*Clin. Microbiol. Rev.*	Bats: Important reservoir hosts of emerging viruses	671
Lau SKP et al.	2005	*Proc. Nat. Acad. Sci. USA*	Severe acute respiratory syndrome coronavirus-like virus in Chinese horseshoe bats	654
Woo PCY et al.	2005	*J. Virol.*	Characterization and complete genome sequence of a novel coronavirus, coronavirus HKU1,from patients with pneumonia	653
Chou KC et al.	2004	*Curr. Med. Chem.*	Structural bioinformatics and its impact to biomedical science	634

**Table 5:** Items and frequency of hot keywords during 2008–2011.

Item	Frequency	Year
PATHOGENESIS	10	2008
NUCLEOCAPSID	10	2008
APOPTOSIS	9	2008
HUMAN METAPNEUMOVIRUS	9	2008
REPLICATION	9	2008
SARS-COV	87	2009
VIRUS	29	2009
CORONAVIRUS	159	2010
INFECTIOUS BRONCHITIS VIRUS	37	2010
PHYLOGENETIC ANALYSIS	26	2010
INFLUENZA	18	2010
RT-PCR	17	2010
MOUSE HEPATITIS VIRUS	9	2011
PIGS	5	2011
PHYLOGENETIC TREE	5	2011

**Table 6:** Top cited papers with over 400 citations during 2008–2011.

Author Name	Year	Publication Journal	Title	GCS
Reyes-Turcu, F.E. et al.	2009	*Ann. Rev. Biochem.*	Regulation and Cellular Roles of Ubiquitin Specific Deubiquitinating Enzymes	782
Ernst, B. et al.	2009	*Nat. Rev. Drug Discov.*	From carbohydrate leads to glycomimetic drugs	480
Ruuskanen, O. et al.	2011	*Lancet*	Viral pneumonia	453
White, J.M. et al.	2008	*Crit. Rev. Biochem. Mol. Biol.*	Structures and mechanisms of viral membrane fusion proteins: Multiple variations on a common theme	445

**Table 7:** Items and frequency of hot keywords during 2012–2018.

Item	Frequency	Year
CHINA	10	2012
RESPIRATORY	8	2012
MOLECULAR EPIDEMIOLOGY	8	2012
EPITOPE	7	2012
MOUSE HEPATITIS VIRUS	6	2012
ENCEPHALOMYELITIS	6	2012
SPIKE GLYCOPROTEIN	6	2012
PATHOGENICITY	6	2012
SARS	60	2013
SPIKE PROTEIN	36	2013
PHYLOGENETIC ANALYSIS	34	2013
FELINE CORONAVIRUS	29	2013
RESPIRATORY VIRUS	26	2013
CORONAVIRUS	252	2014
MERS-COV	96	2014
INFECTIOUS BRONCHITIS VIRUS	65	2014
EPIDEMIOLOGY	44	2014
MERS	33	2015
ANTIVIRAL	12	2015
RECOMBINATION	9	2015
RSV	8	2015
BETACORONAVIRUS	8	2015

**Table 8:** Top cited papers with over 400 citations during 2012–2015.

Author Name	Year	Publication Journal	Title	GCS
Zaki AM et al.	2012	*New Engl. J. Med.*	Isolation of a Novel Coronavirus from a Man with Pneumonia in Saudi Arabia	1308
Assiri A et al.	2013	*New Engl. J. Med.*	Hospital Outbreak of Middle East Respiratory Syndrome Coronavirus	551
Chen Y et al.	2013	*Lancet*	Human infections with the emerging avian influenza A H7N9 virus from wet market poultry: clinical analysis and characterisation of viral genome	546
Raj VS et al.	2013	*Nature*	Dipeptidyl peptidase 4 is a functional receptor for the emerging human coronavirus-EMC	470

**Table 9:** Items and frequency of hot keywords during 2012–2018.

Item	Frequency	Year
SWINE	14	2016
DOG	11	2016
DIAGNOSTICS	8	2016
RESPIRATORY DISEASE	8	2016
DIVERSITY	6	2016
HUMAN CORONAVIRUSES	6	2016
IMMUNOGENICITY	6	2016
INFECTIOUS BRONCHITIS VIRUS (IBV)	6	2016
MIDDLE EAST RESPIRATORY SYNDROME	78	2017
PORCINE EPIDEMIC DIARRHEA VIRUS	74	2017
SARS-COV	47	2017
VACCINE	47	2017
BATS	32	2017
INFECTIOUS BRONCHITIS VIRUS	87	2018
MERS	84	2018
SAUDI ARABIA	61	2018
VIRUS	60	2018
PEDV	50	2018
FELINE INFECTIOUS PERITONITIS	22	2019
ZOONOSIS	21	2019
MOLECULAR EPIDEMIOLOGY	12	2019
RSV	10	2019
3C-LIKE PROTEASE	9	2019
MONOCLONAL ANTIBODY	9	2019

**Table 10:** Top cited papers with over 100 citations during 2016–2019.

Author Name	Year	Publication Journal	Title	GCS
Thompson BT et al.	2017	*New Engl. J. Med.*	Acute Respiratory Distress Syndrome	168
Warren TK et al.	2016	*Nature*	Therapeutic efficacy of the small molecule GS-5734 against Ebola virus in rhesus monkeys	144
de Wit E et al.	2016	*Nat. Rev. Microbiol.*	SARS and MERS: recent insights into emerging coronaviruses	131
Chan JFW et al.	2016	*J. Infect.*	Zika fever and congenital Zika syndrome: An unexpected emerging arboviral disease	115
Sabir JSM et al.	2016	*Science*	Co-circulation of three camel coronavirus species and recombination of MERS-CoVs in Saudi Arabia	113

**Table 11:** Summary of word cloud and co-occurrence maps for five time series.

Time Period	Top 5 Author Keywords	Top 3 Cluster in Cooccurrence Map
2000–2003	- Coronavirus (168) - Mouse hepatitis virus (137) - Identification (90) - Murine coronavirus (82) - Acute respiratory syndrome (71)	- Red cluster (protein and gene) - Yellow cluster (mouse hepatitis) - Blue cluster (SARS and patient cases study)
2004–2007	- Acute respiratory syndrome (718) - Coronavirus (518) - Identification (477) - SAR Scoronavirus (335) - Virus (322)	- Red cluster (Pneumonia and hospital patient) - Green cluster (virus structure, interaction and replication) - Blue cluster (s protein and mouse study)
2008–2011	- Coronavirus (159) - SARS Cov (88) - SARS (74) - Severe acute respiratory syndrome (39) - Infectious bronchitis virus (37)	- Red cluster (protein structure and interact mechanism) - Green cluster (patient diagnosis and sample) - Blue cluster (immune response and t cell study on mouse)
2012–2015	- Coronavirus (252) - MERS Cov (96) - Infectious bronchitis virus (65) - SARS (60) - Epidemiology (44)	- Red cluster (Cell, protein study test on mouse) - Green cluster (respiratory virus patient study) - Blue cluster (IBV and Phylogenetic analysis of bat, cat and pig)
2016–2019	- Coronavirus (384) - MERSCov (245) - Infectious bronchitis virus (87) - Porcine epidemic diarrhea virus (74) - Influenza (64)	- Red cluster (Cell replication and neutralizing antibody) - Green cluster (Respiratory virus and patient) - Blue cluster (IBV, PEDV and phylogenetic analysis)

**Table 12:** Key information of the 2020 literature search.

Period	2020 January to March
Number of publications	384
Source journals	155
Author keywords	630
Average citations per documents	1.81
Authors of single-authored documents	57
Authors of multi-authored documents	1955
Collaboration Index	6.5

**Table 13:** Top cited papers with over 40 citations of 2020.

Author Name	Year	Publication Journal	Title	GCS
Huang CL et al.	2020	*Lancet*	Clinical features of patients infected with 2019 novel coronavirus in Wuhan, China	97
Zhu N et al.	2020	*New Engl. J. Med.*	A Novel Coronavirus from Patients with Pneumonia in China, 2019	68
Chan JFW et al.	2020	*Lancet*	A familial cluster of pneumonia associated with the 2019 novel coronavirus indicating person-to-person transmission: a study of a family cluster	54
Chen NS et al.	2020	*Lancet*	Epidemiological and clinical characteristics of 99 cases of 2019 novel coronavirus pneumonia in Wuhan, China: a descriptive study	42
Zhou P et al.	2020	*Nature*	A pneumonia outbreak associated with a new coronavirus of probable bat origin	41
